# Postural control responses during single-leg balance in individuals with chronic ankle instability under graded visual and somatosensory disruption

**DOI:** 10.3389/fpsyg.2026.1892350

**Published:** 2026-08-13

**Authors:** Seung-Hee Nam, Min-Ju Jeong, Kyoung Jae Kim, Hyun Chul Jung, Kyung-Min Kim

**Affiliations:** 1 Department of Sport Science, Sungkyunkwan University, Suwon-si, Republic of Korea; 2 EVA & Environmental Physiology Laboratory, NASA Johnson Space Center/KBR, Houston, TX, United States; 3 Department of Sports Coaching, Kyung Hee University, Yongin-si, Republic of Korea

**Keywords:** ankle sprains, postural stability, sensorimotor control, sensory reweighting, single-leg balance

## Abstract

**Background:**

Chronic ankle instability (CAI) is a common sequela of lateral ankle sprain and is associated with recurrent injury, persistent functional limitations, sensorimotor deficits, and impaired postural control. However, how individuals with CAI adapt to graded and combined sensory feedback disruption remains unclear. This study aimed to determine whether individuals with CAI exhibit altered postural control during single-leg stance across graded visual, somatosensory, and combined visual-somatosensory disruption compared with healthy controls.

**Methods:**

Fifty-two participants (26 CAI, 26 healthy controls) performed single-leg balance tasks on a force plate under seven sensory conditions: one baseline condition and two graded conditions for each of three sensory disruption types: visual, somatosensory, and combined visual-somatosensory disruption conditions. Balance performance was assessed using center-of-pressure (COP) measures and quantified as percent change from baseline. Separate 2 × 2 mixed-design ANOVAs were conducted for each sensory disruption type and COP outcome.

**Results:**

Significant group-by-level interactions were observed under visual disruption for total COP velocity (*F* = 4.886, *p* = 0.032) and mediolateral COP velocity (*F* = 6.420, *p* = 0.014). Under lower-occlusion visual disruption, the CAI group showed greater postural deterioration than controls, whereas no between-group difference was observed under higher-occlusion visual disruption. No significant group-by-level interactions were found under somatosensory or combined visual-somatosensory disruption. However, under somatosensory disruption, the CAI group showed greater overall deterioration than controls for COP area, total COP velocity, and mediolateral COP velocity. Under combined disruption, both groups showed larger balance declines, with greater deterioration in the CAI group than in controls for total and mediolateral COP velocity.

**Conclusion:**

Individuals with CAI demonstrated greater postural deterioration than healthy controls under several disrupted sensory conditions, while retaining graded responses to somatosensory and combined disruption. The visual disruption findings suggest that individuals with CAI may be particularly vulnerable when visual feedback is partially degraded. These findings may provide a preliminary rationale for informing the development of sensory-targeted rehabilitation strategies aimed at improving functional stability, although future studies using more controlled visual manipulations are warranted.

## 1 Introduction

Lateral ankle sprains (LAS) are among the most common musculoskeletal injuries of the lower extremity, particularly in physically active populations (Waterman et al., 2010; Hootman et al., 2007). Approximately two million individuals in the United States seek treatment for LAS annually, and the associated annual medical costs exceed $1.5 billion (Feger et al., 2017). Despite their high prevalence and burden on healthcare costs, individuals with LAS often do not seek medical care because of the common misperception that this injury is minor (McKay et al., 2001). Consequently, 30 to 74% of individuals with LAS experience residual physical disability (Anandacoomarasamy and Barnsley, 2005; Hertel, 2008; Yeung et al., 1994), and approximately 40% progress to chronic ankle instability (CAI) (Doherty et al., 2016), a condition characterized by recurrent ankle sprains, persistent sensations of the ankle “giving way,” and postural control impairments (Hertel, 2002; Hertel and Corbett, 2019). From a population health perspective, this progression is important because CAI is associated with persistent symptoms, functional limitations, reduced health-related quality of life, recurrent injury, and an increased risk of early post-traumatic ankle osteoarthritis (Gribble et al., 2016; Herzog et al., 2019). Accordingly, LAS and CAI should be considered not only individual clinical or sports-related problems but also important and potentially modifiable public health concerns that require prevention, timely rehabilitation, and long-term management strategies (Hertel and Corbett, 2019; Lee S. et al., 2022; Liu et al., 2025; Liu et al., 2022). Rehabilitation for CAI is typically intended to restore ankle function and stability and to facilitate a return to daily activities and sports participation (Mettler et al., 2015; Gebel et al., 2018; Lesinski et al., 2015). However, despite these efforts, the risk of recurrent ankle sprain remains elevated (Song et al., 2018).

According to the recently updated CAI model, CAI involves three primary impairments: patho-mechanical, sensory-perceptual, and motor-behavioral (Hertel and Corbett, 2019). Among these, postural control deficits are consistently recognized as a primary contributing factor (Hertel, 2008; Hertel, 2002; Tropp et al., 1985; Arnold et al., 2009; Munn et al., 2010; Wikstrom et al., 2009), and are strongly associated with patient-reported instability and an increased risk of recurrent LASs (Kosik et al., 2017). This makes postural control a clinically relevant and potentially modifiable target for rehabilitation and recurrent-injury prevention. Traditionally, impaired postural control has been attributed to proprioceptive deficits caused by damage to ligamentous mechanoreceptors following an initial LAS (Hertel, 2002). One specific mechanism proposed is articular deafferentation, in which mechanoreceptor damage disrupts somatosensory input to the central nervous system (CNS) (Freeman et al., 1965), impairing the ability to obtain accurate sensory feedback necessary for postural control (Sugimoto et al., 2024). However, postural control deficits have also been observed in the contralateral, uninjured limb (Hertel, 2008; Kim, 2020), and regional anesthesia has failed to consistently replicate postural control impairments (Feuerbach et al., 1994; Hertel et al., 1996). These results suggest that impaired postural control in individuals with CAI involves not only proprioceptive deficits but also altered central processing and integration of sensory information (Hertel, 2008; Kim et al., 2017a).

Altered sensory reweighting may be one of the central mechanisms underlying persistent postural control deficits in CAI. Sensory reweighting refers to the CNS’s ability to adjust the relative reliance on visual, vestibular, and somatosensory inputs according to task demands, environmental conditions, and the reliability of available sensory feedback (Peterka, 2018; Peterka, 2002; Assländer and Peterka, 2014). When the reliability of one or more sensory inputs is reduced, the CNS must shift reliance toward more dependable sensory information to maintain balance (Peterka, 2018). In individuals with CAI, ligamentous and mechanoreceptor damage after LAS may reduce the accuracy of somatosensory feedback from the injured ankle, which may increase reliance on visual information as a compensatory strategy (Hertel, 2002; Freeman et al., 1965; Sugimoto et al., 2024). This compensation may become insufficient when visual feedback is also reduced, delayed, or intermittently disrupted in visually challenging environments, such as team-based or opponent-driven activities (Kim, 2020; Song et al., 2016; Han et al., 2022). Therefore, evaluating postural responses under graded visual and somatosensory disruption may help clarify how altered sensory organization contributes to persistent postural control deficits in CAI. Clarifying these mechanisms is also relevant to public health-oriented rehabilitation because sensory-reweighting deficits may represent a modifiable pathway linking ankle injury to recurrent instability and persistent functional limitation.

In addition to sensory-specific difficulty, it is also necessary to determine how postural control changes when combined sensory systems are challenged simultaneously. In daily life, postural control is often maintained under conditions in which visual and somatosensory information are concurrently degraded rather than disrupted in isolation (Nashner, 1982). Evidence from sensory organization research suggests that combined sensory disruption may induce greater instability than isolated disruption (Peterka, 2002), yet this issue remains insufficiently examined in individuals with CAI. Most previous studies have relied on simplified, binary manipulations such as eyes open versus eyes closed (Song et al., 2016; Esteves et al., 2022; Xue et al., 2024; McKeon and Hertel, 2008) or stable versus unstable surfaces (Piri et al., 2025; Esteves et al., 2022), which do not adequately reflect the graded and combined sensory challenges encountered during functional activities. Thus, current evidence remains limited in explaining how individuals with CAI adapt when visual and somatosensory information are disrupted across graded levels and in combination. This approach may provide further insight into how individuals with CAI respond to multi-sensory disruption and may inform sensory integration interventions aimed at restoring functional stability and reducing recurrent instability.

Therefore, the purpose of this study was to determine whether individuals with CAI exhibit altered postural control during single-leg stance across graded levels of visual and somatosensory disruption, both individually and in combination. Single-leg stance was used for this purpose because it is a demanding and commonly used task for detecting postural control deficits in the CAI literature and may be more sensitive than conventional double-leg sensory organization assessments to the multidirectional and task-specific impairments that characterize CAI. Indeed, recent studies have shown that demanding single-leg balance tasks are useful for identifying the multidirectional postural-control deficits and sensorimotor impairments characteristic of individuals with CAI (Xue et al., 2024; De Maio et al., 2026). We hypothesized that individuals with CAI would demonstrate limited adaptability to changes in sensory input—showing minimal performance differences between low and high disruption levels—whereas healthy controls would show progressively greater balance impairments as disruption increased. These differences were anticipated to be most pronounced under the combined visual-somatosensory disruption condition. By clarifying how individuals with CAI adapt to graded and combined sensory disruption, this study may contribute to the development of more targeted rehabilitation and injury-prevention strategies for reducing recurrent ankle sprains and persistent functional disability.

## 2 Materials and methods

### 2.1 Study design

This study employed a cross-sectional design to examine postural control responses in individuals with CAI during single-leg stance under systematically manipulated visual and somatosensory feedback conditions. This approach was based on previous studies that have quantified postural control adaptability by measuring declines in postural stability when sensory feedback is altered from a normal to a perturbed condition (Kim, 2020; Song et al., 2016; Kim et al., 2017b; Song and Wikstrom, 2020). The degree of stability loss under each condition serves as an indicator of the adaptability of the postural control system to changes in the amount or reliability of sensory information (Peterka, 2002).

**Figure 1:**
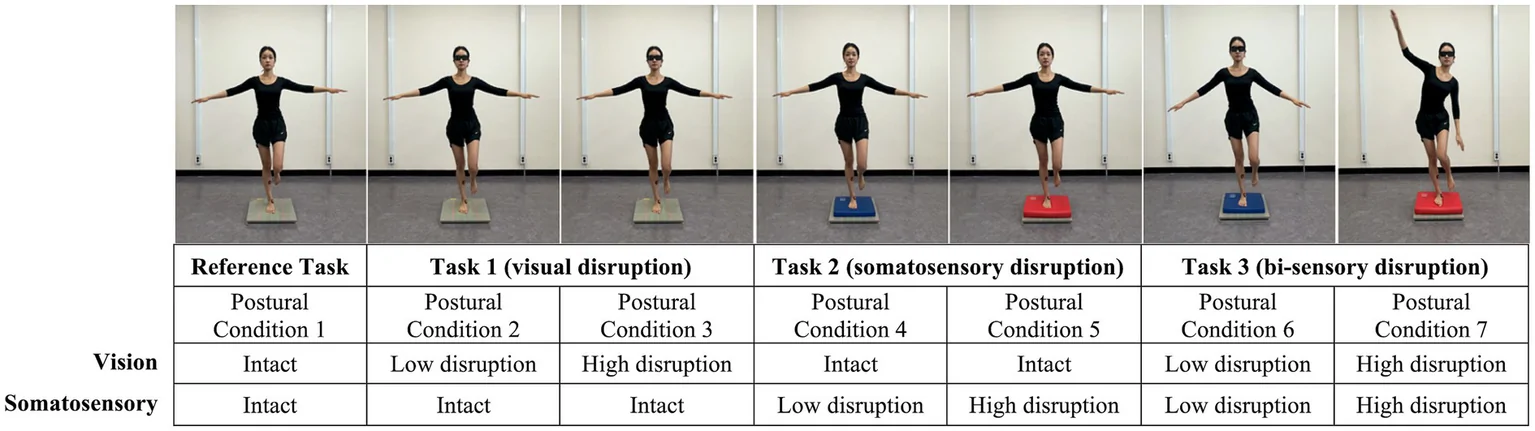
Single-leg balance tests under different levels of sensory conditions. Condition 1: normal vision on a firm surface; conditions 2 and 3: visual disruption using SV2 (5 Hz; 50% occlusion) and SV6 (1.75 Hz; 82.5% occlusion), respectively; conditions 4 and 5: somatosensory disruption using the Airex Solid (110 kg/m^3^) and Cloud (60 kg/m^3^) pads, respectively; and conditions 6 and 7: SV2–Solid and SV6–Cloud combinations, respectively.

The outcome variables were percent changes in center-of-pressure (COP)-based measures of single-leg balance performance from baseline to each sensory disruption condition, including COP area, total COP velocity, anteroposterior COP velocity, and mediolateral COP velocity. Sensory disruptive conditions were classified into three types: vision, somatosensory, and combined vision and somatosensory. For each sensory type, two levels of disruption (low and high) were applied to increase sensory disruptive demands, resulting in a total of seven sensory conditions (Figure 1).

An *a priori* sample size calculation was performed using G*Power (version 3.1.9.6; F tests, ANOVA: repeated-measures, within-between interaction). The expected effect size was derived from a comparable study examining postural control responses under visual-disruption conditions during single-leg stance in individuals with and without CAI (Miao et al., 2024). That study reported group-by-condition interaction effect sizes ranging from *f* = 0.20 to 0.26 across outcome measures; we therefore conservatively adopted the lower bound (*f* = 0.20), which corresponded to the Y-Balance Test. Because the reference study did not report the correlation between repeated measures, we assumed a moderate correlation of *r* = 0.50. The nonsphericity correction was set to 1.00 because the within-subject factor had only two levels. With *α* = 0.05, power (1−*β*) = 0.80, two groups, and two repeated-measure levels, the analysis indicated that a minimum total sample size of 52 participants (26 per group) was required. The present study enrolled 52 participants (26 per group), thereby meeting this requirement.

### 2.2 Participants

We recruited participants from the university community through online recruitment posters, emails and advertisements. We screened 63 individuals for the CAI group and excluded 37 (FAAM criterion not met, *n* = 21; history of lower-limb surgery, *n* = 6; history of lower-limb fracture, n = 4; no giving-way episode within the previous 6 months, *n* = 1; and recurrent ankle sprain within the previous 3 months, *n* = 5). We screened 54 individuals for the healthy control group and excluded 11 (history of lower-limb surgery, *n* = 5; FAAM score below the prespecified threshold, *n* = 6). Of the remaining 43 eligible individuals, we did not select 17 because they did not meet the matching criteria for the CAI group. Accordingly, we enrolled a total of 52 participants (26 individuals with CAI and 26 healthy controls). Participants with CAI were enrolled first, after which healthy controls were recruited using a group-level approach to achieve a comparable sex distribution and similar age, height, body mass, and Godin Leisure-Time Exercise Questionnaire scores. Inclusion criteria for participants with CAI were based on the following: (1) a history of at least one significant lateral ankle sprain, with the initial ankle sprain occurring at least 12 months before study enrollment, (2) at least two episodes of ankle “giving way” within the last 6 months, (3) self-reported perceived ankle instability score less than 24 on the Cumberland Ankle Instability Tool (CAIT), (4) self-assessed ankle disability scores of 90% or less on the Foot and Ankle Ability Measure – Activities of Daily Living (FAAM-ADL), (5) self-assessed ankle disability scores of 80% or less on the FAAM – Sport (FAAM-S). These inclusion criteria of CAI were consistent with the International Ankle Consortium guidelines (Gribble et al., 2013). The healthy controls met the following inclusion criteria: (1) no history of lateral ankle sprains or episodes of ankle instability (e.g., “giving way”), (2) a score of 28 or above on the CAIT, (3) self-assessed ankle disability scores greater than 98% on both the FAAM-ADL and the FAAM-S. For patients with bilateral ankle instability, the limb with the lower scores on the FAAM-ADL and FAAM-S assessments was designated as the involved limb. Exclusion criteria for both groups included: (1) have sustained acute lower extremity or head injuries within the previous 6 months, (2) have known equilibrium disorders (e.g., vestibular dysfunction), (3) have neurological disorders or peripheral neuropathies that could adversely affect single-leg balance, and (4) unable to maintain a single-leg stance for more than 10 s. For healthy controls, the dominant leg was selected based on the leg they preferred for maintaining stability during a one-legged stance, as this leg is typically associated with better postural control (van Melick et al., 2017). This study was approved by the University Institutional Review Board, and informed consent obtained from all participants prior to the study (SKKU 2025-01-029).

### 2.3 Test procedure

All participants attended a single laboratory session in which they completed the single-leg balance tests and a standardized medical history questionnaire. This questionnaire included items regarding their lower extremity medical history, specifically since the onset of lateral ankle sprains, their current physical activity status, the CAIT score to assess the patient-reported ankle instability, and the FAAM, including both the FAAM-ADL and FAAM-S subscales to evaluate ankle function. After completing the questionnaires, demographic and anthropometric (e.g., height and body mass) data were collected. Participants subsequently performed single-leg balance tests under seven sensory conditions as shown in Figure 1. The order of the test conditions was randomized at the task-block level. For each participant, the order of the four task blocks—reference, visual, somatosensory, and combined visual–somatosensory—was determined using a computer-generated random permutation without replacement. The examiner informed each participant of the assigned sequence before testing. Within each sensory-disruption block, the levels were fixed from lower to higher to facilitate safe and successful task completion. Prior to the experimental trials, participants were provided with a 5-min acclimation period to familiarize themselves with the goggles and foam pads. For each condition, participants stood barefoot on an Accusway force platform (AMTI Corp., Watertown, MA, USA) with their arms free for balance and performed three 10-s trials of single-leg stance while focusing on a 15 cm circular visual target placed at eye level 3 meters in front of them. For the somatosensory and combined conditions, a foam pad was placed directly on top of the force platform. Testing was conducted under consistent overhead laboratory lighting. The positions of the arms and non-stance limb, as well as degree of stance-knee flexion, were self-selected. This duration and unrestricted arm movement were selected to quantify COP-based postural control responses across challenging sensory feedback conditions while minimizing fatigue, failed trials, and missing data during repeated single-leg stance testing. After completing the three trials for each condition, a 1-min rest period was given before the next single-limb stance condition protocol. Standardized rest intervals were provided to both groups between repeated trials. A total of 21 trials were conducted, with one to three practice trials allowed for each condition. If a participant was unable to complete a 10-s trial, the data were disregarded, and the trial was conducted again until three successful outcomes were achieved for each condition. Participants were permitted up to 10 failed attempts per condition; testing was discontinued if they could not complete three successful trials within this limit. A trial was classified as a failed attempt and was repeated if the participant: (a) touched down with the non-involved limb; (b) contacted the stance limb; (c) hopped or stepped with the stance limb; (d) lifted their forefoot or heel (Terada et al., 2019). Only successful trials were included in the COP analyses. The number of failed trials was recorded separately as an indicator of task difficulty and compared between groups.

### 2.4 Sensory feedback disruption

The experimental conditions were classified into three task categories: visual disruption (Task 1), somatosensory disruption (Task 2), and combined disruption (Task 3). Condition 1 served as the reference (baseline, condition 1) and involved no sensory perturbation, performed with eyes open on a firm surface. Visual perturbations were applied using stroboscopic goggles (Senaptec LLC, Beaverton, OR, USA), which intermittently disrupt visual feedback by cycling the lenses between transparent and opaque states without completely blocking vision (Kim et al., 2017b; Kim et al., 2020). The stroboscopic goggles provide eight manufacturer-defined difficulty levels, with the transparent phase fixed at 0.1 s and the opaque phase progressively increasing at higher level settings. Based on previous research (VanDeMark et al., 2021), we selected two distinct visual disruption settings. Specifically, the lower-occlusion strobe level 2 setting (Condition 2, frequency of 5 Hz; duration of transparent lens is 0.1 s; duration of opaque lens is 0.1 s, 50% visual occlusion, SV2) and the higher-occlusion strobe level 6 setting (Condition 3, frequency of 1.75 Hz; duration of transparent lens is 0.1 s; duration of opaque lens is 0.47 s, 82.5% visual occlusion, SV6) were used. These settings were selected based on manufacturer-defined levels and prior research (VanDeMark et al., 2021) but were not validated as distinct levels of visual-disruption severity. Somatosensory input was disrupted by placing foam pads (Balance-pad Airex®, Airex AG, Sins, Switzerland) over the force plate (Mademli et al., 2021), using a firmer pad for low-level disruption (Condition 4, Airex Balance Pad-Solid, raw density 110 kg/m^3^) and a softer pad for high-level disruption (Condition 5, Airex Balance Pad-Cloud, raw density 60 kg/m^3^) each performed under normal visual conditions (Song and Wikstrom, 2020; Shumway-Cook and Horak, 1986). This graded manipulation of surface stability reduces the fidelity of plantar and ankle joint mechanoreceptor feedback, thereby challenging the sensory reweighting process in postural control (Peterka, 2002). Finally, two combined sensory conditions were included. Condition 6 combined the SV2 setting with the firmer foam pad, whereas condition 7 combined the SV6 setting with the softer foam pad. In summary, condition 1 served as the baseline condition and involved standing on a firm surface with normal vision. Conditions 2 and 3 involved SV2 and SV6 visual conditions, respectively. Conditions 4 and 5 involved low- and high-level somatosensory disruption, respectively. Conditions 6 and 7 involved the SV2-firmer foam and SV6-softer foam combinations, respectively, as shown in Figure 1. The protocol was designed to systematically induce sensory conflict by altering visual and/or somatosensory input.

### 2.5 Data processing

For each test trial, COP data were recorded using an Accusway force platform (AMTI Corp., Watertown, MA, USA) at a sampling rate of 100 Hz. The recorded COP data were filtered with a fourth-order, zero-lag, low-pass Butterworth filter with a cutoff frequency of 5 Hz. COP metrics were computed using custom-made MATLAB software (MATLAB; The MathWorks Inc., Natick, MA, USA). COP data from the foam conditions were processed identically to those from the firm-surface conditions.

Four COP-based parameters were calculated: total area, total velocity, its segmental velocities in anteroposterior (AP) and mediolateral (ML) direction. Consistent with previous research (Palmieri et al., 2002), total area (cm^2^) was calculated as the convex-hull area enclosing the outermost points of the COP trajectory, with larger areas suggesting poorer postural control (Nam and Kim, 2025). Total velocity (cm/s) was calculated as the total two-dimensional COP path length divided by trial duration, where higher velocities suggest more frequent or rapid corrective movements. In addition, AP and ML velocities (cm/s) were calculated as the total COP displacement in the respective directions divided by trial duration. These measures capture the directional speed of COP movement and serve as indicators of balance performance in each plane (Palmieri et al., 2002).

All outcome variables were averaged across three trials for each condition. Consistent with previous research (Kim, 2020), this study used the following formula to calculate the percent change in unipedal postural control from the normal sensory condition to each disrupted condition, representing the reduction in single-leg balance performance when transitioning from the normal condition to disrupted condition. Percent change was calculated relative to the baseline condition. Higher positive values indicated a greater increase in COP-based sway or velocity from baseline and were interpreted as greater deterioration in postural control.

$$(\frac{\text{Sensory disrupted condition}-\text{Sensory normal condition}}{\text{Sensory normal condition}})\times 100$$

For descriptive contextualization of the overall postural-control response pattern, we calculated *post hoc*, for each group and sensory condition, the equally weighted arithmetic mean of the group-level mean percentage changes across four COP measures: total COP area, total COP velocity, mediolateral COP velocity, and anteroposterior COP velocity. We used these values only as exploratory descriptive summaries.

### 2.6 Statistical analysis

Independent t-tests were performed to compare the demographic data between the individuals with CAI and healthy control groups. We conducted a separate 2 × 2 (group-by-sensory disruption level) analysis of variance (ANOVA) with repeated measures to determine significant differences between groups (CAI and Control) and sensory disrupted levels (low and high) on single leg balance performance, as quantified by COP-based parameters. When significant interactions were found, *post hoc* analyses included independent *t*-tests (between groups) and paired *t*-tests (within groups), with Bonferroni corrections applied to adjust for multiple comparisons. Because no single primary outcome was designated and no multiplicity adjustment was applied across the separate ANOVA models, these analyses were treated as exploratory. To examine whether the primary findings were robust after adjustment for potential confounding variables, covariate-adjusted sensitivity analyses were additionally performed using repeated-measures general linear models. Age, height, body mass, and physical activity level, specified *a priori* as measured confounders were entered as continuous covariates. Sex was not entered as a covariate because sex distribution was identical between groups. Additionally, Cohen’s d effect sizes and associated 95% confidence intervals (CIs) were calculated to assess the magnitude of the differences, with values interpreted as follows: > 0.2 as small, 0.5 as moderate, and 0.8 as large (Cohen, 1988). All statistical analyses were conducted using SPSS 29.0 (IBM Corp, Armonk, NY, USA) with the alpha level set at 0.05.

## 3 Results

### 3.1 Participant demographics

A total of 52 participants were included in this study, comprising 26 individuals with CAI (15 males and 11 females) and 26 healthy participants (15 males and 11 females) with no history of lateral ankle sprains. All participants attempted single-leg balance tasks under seven sensory disruption conditions. No significant group differences were found in age, height, body mass, or physical activity level by Godin Leisure-Time scores (*p >* 0.05). However, the CAI group showed significantly lower scores on the CAIT, FAAM-ADL, and FAAM-S compared to the control group (all *p* ≤ 0.004). The CAI group also demonstrated a significantly greater number of failed trials than the control group, indicating greater difficulty completing the single-leg balance tasks under sensory disruption conditions. One participant in the CAI group was excluded from analysis under the high-level combined visual-somatosensory disruption condition (Condition 7) due to being unable to maintain a single-leg stance for 10 s. Accordingly, the visual and somatosensory analyses included all 52 participants (CAI: *n* = 26; controls: *n* = 26), whereas the complete-case analysis of the combined visual–somatosensory conditions included 51 participants (CAI: *n* = 25; controls: *n* = 26). The descriptive data of participant demographics are presented in Table 1.

**Table 1:** Participant demographics.

Group	CAI group (*n* = 26)	Healthy control group (*n* = 26)	*p*-value	Group effect size^a^
Sex	15 males, 11 females	15 males, 11 females	1.000	—
Age (years)	22.6 ± 4.7	22.6 ± 2.6	0.685	0.11 (−0.43, 0.66)
Height (cm)	171.9 ± 10.5	171.0 ± 8.4	0.734	0.09 (−0.45, 0.64)
Body mass (kg)	73.3 ± 14.3	66.2 ± 11.1	0.054	0.54 (−0.01, 1.10)
Years since the first significant ankle sprain	7.8 ± 5.8	N/A	—	—
Number of previous ankle sprains	6.9 ± 5.2	N/A	—	—
Months since the latest ankle sprain	15.5 ± 17.4	N/A	—	—
Number of ankles giving way within 6 months	16.8 ± 18.5	N/A	—	—
Pain visual analog scale	3.12 ± 2.18	N/A	—	—
Laterality of CAI	20 bilateral, 6 unilateral	N/A	—	—
Tested limb	Involved limb: 11 right, 15 left	Dominant limb: 10 right, 16 left	—	—
CAIT	12.1 ± 3.5	29.7 ± 0.7	<0.001*	−7.34 (−8.87, −5.80)
FAAM-ADL (%)	84.3 ± 7.5	99.8 ± 0.5	<0.001*	−2.93 (−3.76, −2.17)
FAAM-Sport (%)	63.8 ± 12.6	99.7 ± 1.3	<0.001*	−4.06 (−5.01, −3.09)
Godin Leisure-Time Exercise	30.6 ± 19.9	30.3 ± 20.5	0.967	0.01 (−0.53, 0.55)
Number of failed trials^b^	8.5 ± 10.1	2.3 ± 3.3	0.004*	0.83 (0.26, 1.40)

ADL, activity of daily living; CAI, chronic ankle instability; CAIT, Cumberland Ankle Instability Tool; FAAM, Foot and Ankle Ability Measure; N/A; not applicable.

Values are presented as mean ± standard deviation.

^*^Indicates a significant group difference (*p* < 0.05).

a Cohen’s d estimate of effect size was calculated between two groups using pooled standard deviation, along with its associated 95% confidence interval.

b Defined as trials in which participants failed to maintain balance for at least 10 s; values represent the total number of failed trials accumulated across all sensory conditions.

### 3.2 Visual feedback disruption

**Figure 2:**
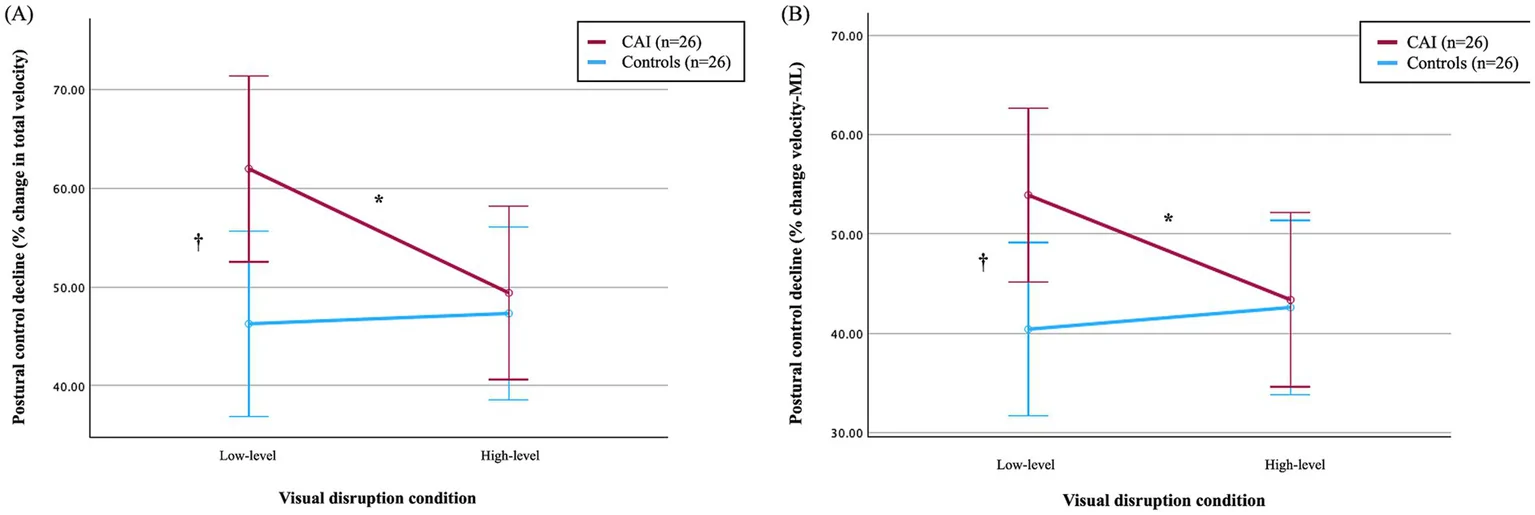
Group-by-sensory disruption level interaction in postural control decline under visual disruption conditions. Significant group-by-level interactions were observed in total velocity **(A)** and velocity-ML **(B)** (*p* < 0.05). Data are presented as estimated marginal means, with error bars representing 95% confidence intervals.*A significant within-group difference, the CAI group indicated significantly greater postural control decline under the low-level than high-level visual disruption (*p* < 0.05). ^†^Under the low-level visual condition, the CAI group exhibited significantly greater postural control decline than the control group (*p* < 0.05).

Significant group-by-sensory disruption level interactions were found in two of the four COP-based parameters: total velocity (*F* = 4.886, *p* = 0.032) and velocity-ML (*F* = 6.420, *p* = 0.014) shown in Table 2. For total velocity, *post hoc* analyses revealed that under the lower-occlusion visual disruption (SV2), the CAI group exhibited significantly greater postural control decline (62.0%) compared to the control group (46.3%) with moderate effect sizes (*d* = 0.66, 95% CIs [0.10, 1.21]). Similar findings were also observed for velocity-ML (CAI: 53.9% vs. controls: 40.4%, *d* = 0.61, 95% CIs [0.05, 1.16]). No significant group differences were observed under the higher-occlusion visual disruption (SV6). Within the CAI group, postural control showed a significantly greater balance decline under the low-level than the high-level visual disruption (*p* < 0.05), whereas no such difference was found in the control group (Figure 2). No significant interaction or group main effect was found for total area and velocity-AP. No significant main effects of visual disruption level were observed for any COP outcomes (all *p* > 0.05).

**Table 2:** Postural control decline during single-leg stance under low (SV2) and high (SV6) levels of visual disrupted feedback.

COP parameters	Percent change (%)			Group-by-VIS interaction			Group main effect			VIS main effect			Group effect size^b^
	VIS level	Chronic ankle instability (*n* = 26)	Healthy control (*n* = 26)	*F*_(1,50)_	*p*	η_p_^2^^a^	*F*_(1,50)_	*p*	η_p_^2^^a^	*F*_(1,50)_	*p*	η_p_^2^^a^	
Total Area (%)	Low High	135.11 ± 72.34 110.63 ± 64.55	117.19 ± 73.17 119.79 ± 62.68	1.779	0.188	0.034	0.075	0.786	0.001	1.163	0.286	0.023	0.25 (−0.30, 0.79) −0.14 (−0.69, 0.40)
Total velocity (%)	Low High	61.97 ± 25.91 49.39 ± 22.64	46.26 ± 21.70 47.31 ± 21.92	4.886	0.032^*^	0.089	2.504	0.120	0.048	3.497	0.067	0.065	0.66 (0.10, 1.21) 0.09 (−0.45, 0.63)
Velocity-ML (%)	Low High	53.89 ± 21.89 43.36 ± 22.57	40.39 ± 22.42 42.59 ± 21.97	6.420	0.014^*^	0.114	1.609	0.211	0.031	2.854	0.103	0.052	0.61 (0.05, 1.16) 0.04 (−0.51, 0.58)
Velocity-AP (%)	Low High	69.57 ± 34.21 55.66 ± 29.05	53.05 ± 26.24 52.98 ± 25.87	3.053	0.087	0.058	1.875	0.177	0.036	3.113	0.084	0.059	0.54 (−0.02, 1.09) 0.10 (−0.45, 0.64)

AP, anteroposterior; COP, center of pressure; ML, mediolateral; VIS, visual disruption conditions; SV2, stroboscopic level 2; SV6, stroboscopic level 6.

Values are presented as mean ± standard deviation.

^*^Indicates a significant interaction between group and levels of visual disruption (*p* < 0.05).

a Partial eta-squared represents the effect size for each ANOVA effect and was interpreted as small (0.01), moderate (0.06), or large (0.14).

b Cohen’s d estimate of effect size was calculated for between two groups using the pooled standard deviation and is presented with its 95% confidence interval. Values of 0.2, 0.5, and 0.8 were interpreted as small, moderate, and large effect sizes, respectively.

### 3.3 Somatosensory feedback disruption

**Figure 3:**
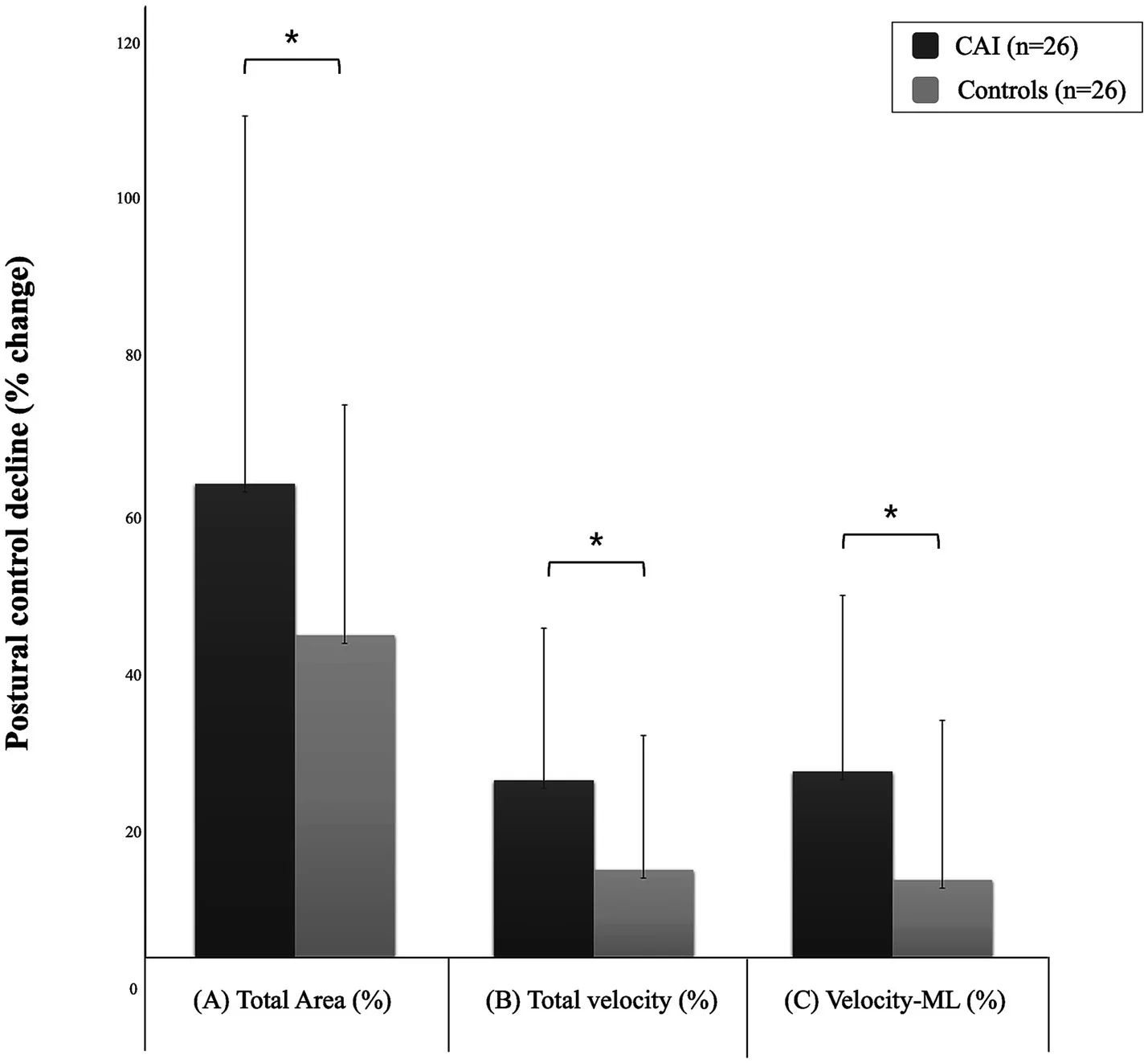
Group differences in % change in postural control decline under somatosensory disruption conditions for **(A)** total area, **(B)** total velocity, **(C)** velocity-ML. Bars represent estimated marginal means averaged across the low- and high-level disruption conditions, with error bars representing 95% confidence intervals. *Indicate a significant main group effect (*p* < 0.05).

There were no significant group-by-sensory disruption level interactions for all COP-based parameters, as shown in Table 3. However, we found significant group main effects for all four COP measures except the Velocity-AP: total area (*F* = 4.357, *p* = 0.042), total velocity (*F* = 6.009, *p* = 0.018), and velocity-ML (*F* = 6.259, *p* = 0.016). Across these parameters, the CAI group consistently showed greater deterioration in postural control than the control group regardless of the somatosensory disruption levels (Figure 3). The group differences appeared to be moderate (*d* = 0.48 to 0.71). For the velocity-AP measure, there was neither significant interaction (*F* = 1.540, *p* = 0.220) nor group main effect (*F* = 2.904, *p* = 0.095). Significant main effects of somatosensory-disruption level were observed for all COP outcomes (all *p* < 0.05).

**Table 3:** Postural control decline during single-leg stance under low and high levels of somatosensory disrupted feedback.

COP parameters	Percent change (%)			Group-by-SOM interaction			Group main effect			SOM main effect			Group effect size^d^
	SOM level	Chronic ankle instability (*n* = 26)	Healthy control (*n* = 26)	*F*_(1,50)_	*p*	η_p_^2^^c^	*F*_(1,50)_	*p*	η_p_^2^^c^	*F*_(1,50)_	*p*	η_p_^2^^c^	
Total Area (%)	Low High	46.74 ± 41.20 72.87 ± 51.10	29.28 ± 30.20 51.96 ± 27.94	0.096	0.758	0.002	4.357	0.042^a^	0.080	19.250	<0.001^b^	0.278	0.48 (−0.07, 1.03) 0.51 (−0.05, 1.06)
Total velocity (%)	Low High	13.56 ± 17.02 31.15 ± 21.05	5.21 ± 14.56 16.89 ± 19.21	2.176	0.146	0.042	6.009	0.018^a^	0.107	53.491	<0.001^b^	0.517	0.53 (−0.03, 1.08) 0.71 (0.14, 1.27)
Velocity-ML (%)	Low High	14.09 ± 18.95 32.73 ± 25.19	3.87 ± 15.64 15.75 ± 23.87	2.181	0.146	0.042	6.259	0.016^a^	0.111	44.452	<0.001^b^	0.471	0.59 (0.03, 1.14) 0.69 (0.13, 1.25)
Velocity-AP (%)	Low High	15.10 ± 26.60 31.41 ± 29.40	7.21 ± 17.96 18.32 ± 17.46	1.540	0.220	0.030	2.904	0.095	0.055	42.726	<0.001^b^	0.461	0.35 (−0.20, 0.89) 0.54 (−0.02, 1.09)

AP, anteroposterior; COP, center of pressure; ML, mediolateral; SOM, somatosensory disruption condition.

Values are presented as mean ± standard deviation.

a Indicates a significant group main effect (*p* < 0.05).

b Indicates a significant main effect of somatosensory disruption level (*p* < 0.05).

c Partial eta-squared represents the effect size for each ANOVA effect and was interpreted as small (0.01), moderate (0.06), or large (0.14).

d Cohen’s d estimate of effect size was calculated for between two groups using the pooled standard deviation and is presented with its 95% confidence interval. Values of 0.2, 0.5, and 0.8 were interpreted as small, moderate, and large effect sizes, respectively.

### 3.4 Combined visual-somatosensory feedback disruption

**Figure 4:**
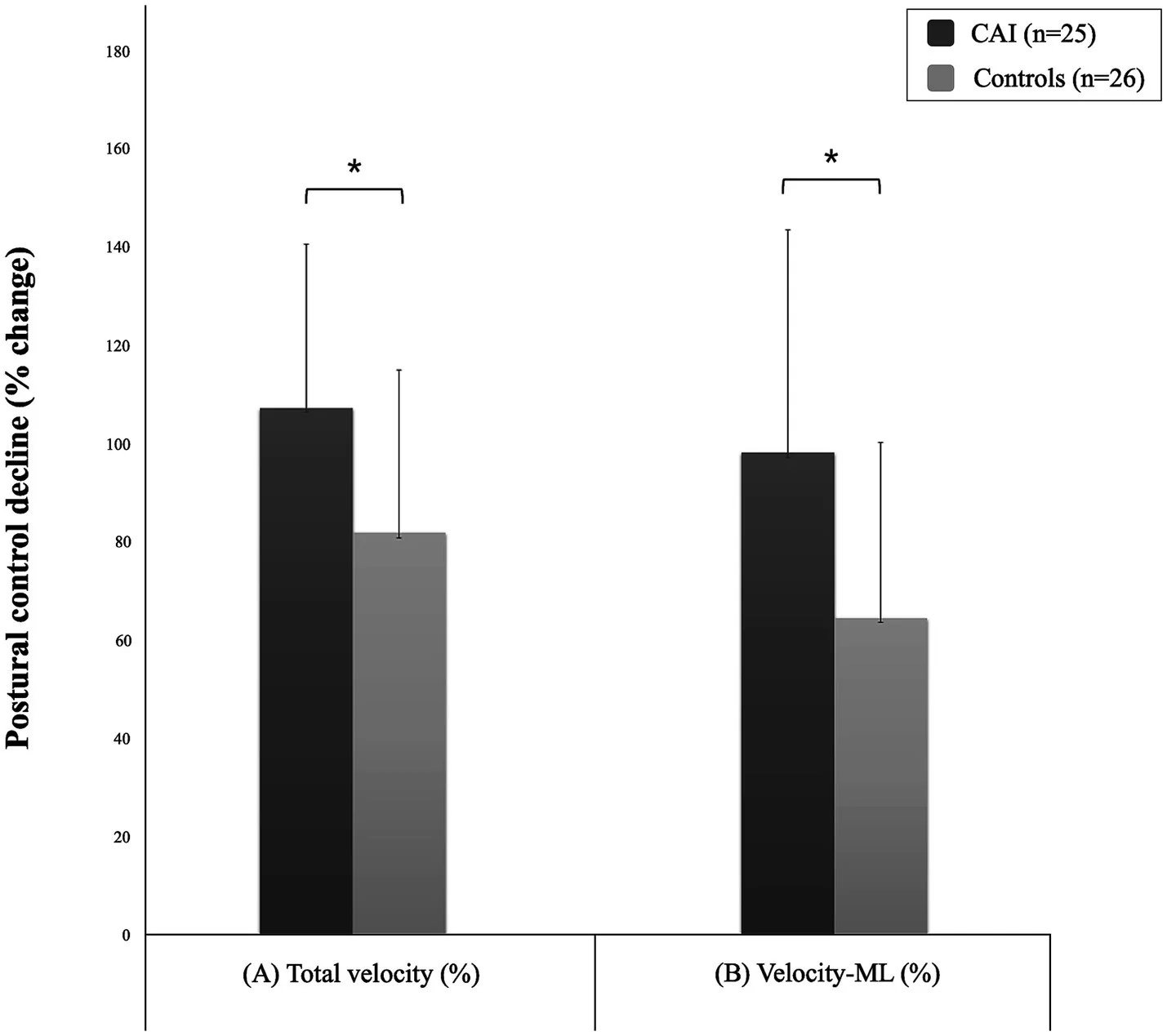
Group differences in % change in postural control decline under combined visual-somatosensory disruption conditions. **(A)** Total velocity; **(B)** Velocity-ML. Bars represent estimated marginal means averaged across the low- and high-level disruption conditions, with error bars representing 95% confidence intervals.*indicate a significant main group effect (*p* < 0.05).

No significant group-by-sensory disruption level interactions were found for any COP-based parameters (Table 4). However, there were significant group main effects for the total velocity (*F* = 7.439, *p* = 0.009) and average velocity in ML direction parameters (*F* = 9.098, *p* = 0.004). The CAI group exhibited significantly greater postural control decline (102.8%) than the control group (75.2%), regardless of the level of sensory disruption (Figure 4). The group differences were moderate to large (*d* = 0.61 to 0.89). No significant group differences were observed in total area (*F* = 0.596, *p* = 0.444) and velocity-AP parameters (*F* = 3.294, *p* = 0.076). Significant main effects of combined visual-somatosensory disruption level were observed for all COP outcomes (all *p* < 0.05). Condition-specific COP values before conversion to percentage changes are presented in Supplementary Table S1.

**Table 4:** Postural control decline during single-leg stance under low and high levels of combined visual-somatosensory disrupted feedback.

COP parameters	Percent change (%)			Group-by-VIS-SOM interaction			Group main effect			VIS-SOM main effect			Group effect size^d^
	VIS-SOM level	Chronic ankle instability (*n* = 25)	Healthy control (*n* = 26)	*F*_(1,49)_	*p*	η_p_^2^^c^	*F*_(1,49)_	*p*	η_p_^2^^c^	*F*_(1,49)_	*p*	η_p_^2^^c^	
Total Area (%)	Low High	234.59 ± 100.04 342.19 ± 141.25	217.50 ± 99.18 31.98 ± 120.12	0.008	0.930	0.000	0.596	0.444	0.012	30.080	<0.001^b^	0.380	0.17 (−0.37, 0.72) 0.17 (−0.38, 0.72)
Total velocity (%)	Low High	91.86 ± 35.24 122.97 ± 52.81	68.66 ± 29.21 95.48 ± 36.55	0.080	0.779	0.002	7.439	0.009^a^	0.132	25.696	<0.001^b^	0.344	0.72 (0.15, 1.28) 0.61 (0.04, 1.17)
Velocity-ML (%)	Low High	80.82 ± 33.63 115.58 ± 57.92	53.52 ± 27.73 83.06 ± 36.50	0.171	0.681	0.003	9.098	0.004^a^	0.157	36.447	<0.001^b^	0.427	0.89 (0.31, 1.45) 0.67 (0.11, 1.24)
Velocity-AP (%)	Low High	103.51 ± 50.06 132.92 ± 60.28	84.88 ± 38.45 109.18 ± 44.93	0.076	0.784	0.002	3.294	0.076	0.063	15.953	<0.001^b^	0.246	0.42 (−0.13, 0.97) 0.45 (−0.11, 1.00)

AP, anteroposterior; COP, center of pressure; ML, mediolateral; VIS-SOM, combined visual-somatosensory disruption condition.

Values are presented as mean ± standard deviation.

a Indicates a significant group main effect (*p* < 0.05).

b Indicates a significant main effect of combined visual-somatosensory disruption level (*p* < 0.05).

c Partial eta-squared represents the effect size for each ANOVA effect and was interpreted as small (0.01), moderate (0.06), or large (0.14).

d Cohen’s d estimate of effect size was calculated for between two groups using the pooled standard deviation and is presented with its 95% confidence interval. Values of 0.2, 0.5, and 0.8 were interpreted as small, moderate, and large effect sizes, respectively.

### 3.5 Covariate-adjusted sensitivity analysis

Covariate-adjusted sensitivity analyses showed that the overall pattern of findings remained consistent with the original 2 × 2 ANOVA results after adjustment for age, height, body mass, and physical activity level. In addition, the group main effect for average COP velocity in the AP direction under combined visual-somatosensory disruption became statistically significant after adjustment. Detailed results are presented in Supplementary Table S2.

## 4 Discussion

### 4.1 Visual feedback disruption

We found that under lower-occlusion visual disruption (SV 2: 50% occlusion), the CAI group showed a 15-percentage point greater decline in balance performance than the control group, whereas no significant between-group difference emerged at higher-occlusion visual disruption (SV 6: 82.5% occlusion). Unexpectedly, the control group showed no significant change in balance decline between lower and higher-occlusion visual disruption. One possible reason is that increasing visual occlusion from 50% to 82.5% may not meaningfully alter visual information enough to further compromise single-leg balance, as similar studies have shown diminishing impacts on postural sway beyond moderate levels of occlusion (Kim et al., 2020; Lee et al., 2022a; Robey et al., 2024).

In contrast, the CAI group demonstrated a significantly greater decline in postural control under lower-occlusion visual disruption (SV2), which is generally consistent with previous findings that individuals with CAI exhibit excessive reliance on visual input (Song et al., 2016; Xue et al., 2024; Knapp et al., 2011). One possible interpretation is that when visual information remains partially available but becomes unreliable, individuals with CAI may continue to over-weight this degraded visual input rather than effectively down-weighting it (Sugimoto et al., 2024). Under lower-occlusion visual disruption (SV2), the remaining visual cue may still be used for postural control yet may not be sufficiently reliable to maintain stable balance, thereby resulting in greater postural control deterioration in the CAI group. By contrast, under higher-occlusion visual disruption (SV6), visual input may have become sufficiently unreliable that individuals with CAI relied less on visual feedback and more on alternative sensory information (Sugimoto et al., 2024; Hu et al., 2026). Importantly, the lack of a between-group difference at this level should not be interpreted as normalization of postural control in CAI. Rather, given the known somatosensory deficits associated with CAI, reduced access to visual information may have encouraged greater reliance on nonvisual sensory inputs, although the present COP outcomes cannot directly determine the underlying compensatory strategy (). This possibility is partly supported by previous sensory organization test (SOT)-based study showing reduced postural movement variability in CAI under visually occluded condition (), although future studies employing non-linear analyses of postural dynamics are needed to clarify these potential compensatory mechanisms. Importantly, these COP-based findings should be interpreted with caution as indirect behavioral evidence of altered sensory reweighting mechanisms, rather than as a direct quantification of the relative weighting of sensory inputs. Nevertheless, from a clinical perspective, these findings suggest that individuals with CAI may be particularly vulnerable in situations where visual information is intermittently degraded or unstable. Therefore, rehabilitation for CAI may benefit from incorporating graded and dynamic visual perturbations, rather than relying solely on abrupt visual deprivation (e.g., eyes-closed balance training), to progressively challenge visual reliance under complex sensory conditions.

Nevertheless, these findings should be interpreted with caution because the unexpected sensitivity observed in the CAI group may also have been influenced by the stroboscopic design: at the lower disruption level, the rapid on–off cycles may have produced a stronger destabilizing flicker effect than anticipated. The higher flicker frequency in the low-level visual disruption condition (SV 2: 5 Hz, 5 flicker cycles per second), which appeared to challenge postural control more than the high-level visual disruption (SV 6: 1.75 Hz, 1.75 flicker cycle per second), may be suboptimal for research manipulating the level of visual feedback. Future studies should employ more tightly controlled visual environments, such as adjustable lighting systems or virtual reality platforms, to manipulate visual input more systematically (Wagner and Merfeld, 2023; Wittstein et al., 2020; Gerber et al., 2024).

### 4.2 Somatosensory feedback disruption

We observed that both groups exhibited a graded decline in single-leg balance performance as somatosensory disruption increased from low (solid-foam pad) to high (cloud-foam pad). Specifically, the control group’s postural control declined by 11% under low-level disruption and further by 26% under high-level disruption. As expected, the greater instability induced by the softer foam pad elicited a significant increase in balance decline, reflecting an expected graded postural response to degraded proprioceptive input. Interestingly, the CAI group showed a similar pattern: balance performance decreased by 22% with low-level disruption and by 42% with high-level disruption, demonstrating a significant incremental decline. This finding contradicts our hypothesis that individuals with CAI, who demonstrate documented proprioceptive deficits following ligament injury (Hertel, 2002; Freeman et al., 1965; McKeon and Hertel, 2008), would fail to adjust their postural responses to increasing somatosensory disruption. Instead, the CAI group showed a clear, graded increase in balance decline from low to high foam instability, suggesting that individuals with CAI may retain some capacity to adjust postural responses as somatosensory reliability decreases (Munn et al., 2010; Song and Wikstrom, 2020). This finding supports previous research using optimal SOT indicating that CAI patients can partially compensate for somatosensory deficits through sensory reweighting mechanisms (Hu et al., 2026). It is important to note, however, that at both low and high disruption levels, the absolute magnitude of postural decline in the CAI group remained markedly higher than in controls (22% vs. 11% and 42% vs. 26%, respectively shown in Table 3), showing that, even though they adapt to increasing instability, individuals with CAI still struggle more overall because of the existing deficits in their ankle proprioception. This result is in line with findings by Piri et al. (2025), who reported that CAI patients exhibit approximately 30% greater reductions in single-leg balance compared to controls. While previous studies have reported conflicting results regarding the effect of somatosensory disruption on postural control (Piri et al., 2025; Esteves et al., 2022; Linens et al., 2014), the current study supports the notion that impaired proprioception in CAI patients consistently leads to greater postural deficits when somatosensory feedback is disrupted. However, the evidence on the effects of somatosensory disruption on postural control remains limited, and the methods used to induce somatosensory perturbations are highly heterogeneous. Therefore, this finding warrants further investigation. Nevertheless, the present findings support the potential value of incorporating targeted proprioceptive training into rehabilitation protocols to address sensory impairments and enhance functional stability under varying surface conditions (De Ridder et al., 2015; Stanek et al., 2013).

### 4.3 Combined visual-somatosensory feedback disruption

When both visual and somatosensory feedback were degraded simultaneously—using SV 2 goggles (50% occlusion) with solid foam and SV 6 goggles (82.5% occlusion) with cloud foam—postural control decline increased in both groups. Healthy controls showed a 105% decline under combined low visual-somatosensory disruption and a 150% decline under combined high visual-somatosensory disruption. Individuals with CAI experienced even larger declines: approximately 130% under combined low-level visual-somatosensory disruption and 180% under high-level disruption, suggesting that concurrent sensory challenges may greater deterioration balance impairments (Piri et al., 2025; Kim et al., 2017b; Hu et al., 2026). Our finding supports previous research reporting that combined disruption of visual and somatosensory input leads to greater balance impairments, particularly under eyes closed condition on a soft surface compared to eyes closed alone, as evidenced by increased postural sway (Piri et al., 2025). These observations reflect the graded balance decline seen with somatosensory-only disruption, but contrast with the visual-only condition, in which group differences appeared only under low-level occlusion. These results suggest that increasing somatosensory disruption may have imposed a greater challenge to single-leg balance than increasing visual disruption in the present protocol. These findings are broadly consistent with a recent SOT study that despite reduced sensory reweighting capacity in CAI patients, somatosensory input may remain an important source for maintaining postural control (Hu et al., 2025). Previous work has also shown that altered surface conditions can substantially influence postural sway, supporting the importance of somatosensory information for balance control (Peterka, 2002; Corbin et al., 2007). In addition to the greater role of somatosensory input over single-leg balance, the combined visual-somatosensory feedback challenges produced even larger balance declines in CAI group, highlighting their heightened vulnerability when multiple sensory systems are taxed (Piri et al., 2025; Kim et al., 2017b; Hu et al., 2026). However, these findings should be interpreted cautiously because the present study did not directly assess visual–somatosensory interactions or the relative contribution of each sensory modality. Future studies using a complete factorial design need to directly examine the independent and interactive effects of visual and somatosensory disruption. Despite these interpretive limitations, the greater deterioration observed in the CAI group under combined sensory disruption suggests that rehabilitation programs may benefit from progressively integrating multisensory balance challenges rather than training single sensory modalities in isolation. For instance, combining stroboscopic vision exercises with balance work on foam surfaces has been shown to further enhance postural stability in individuals with CAI (Kim et al., 2021; Lee et al., 2024; Lee et al., 2022b). Similarly, virtual reality environments may offer a controlled approach for manipulating multiple sensory cues and progressively challenging balance control (Wagner and Merfeld, 2023).

### 4.4 Clinical implications

The present findings suggest that individuals with CAI may exhibit distinct vulnerabilities across sensory challenge conditions, providing a preliminary rationale for considering targeted multisensory rehabilitation approaches. First, CAI group exhibited more sensitive and exaggerated postural control responses to sensory feedback disruption compared to healthy controls. However, under somatosensory and combined disruption conditions, their postural responses still changed across increasing levels of sensory challenge. These findings provide indirect behavioral evidence that sensory organization in individuals with CAI may not be uniformly impaired across all sensory conditions and that progressively modified sensory loading in rehabilitation may warrant further investigation. For instance, traditional balance training protocols often rely on abrupt or extreme sensory challenges—such as transitioning directly from eyes open to eyes closed, or from firm ground to foam pads—without adjusting the level of difficulty (Mettler et al., 2015; Hall et al., 2018; Mollà-Casanova et al., 2021). Such conditions may impose a substantial task difficulty for some individuals with CAI, preventing proper execution of the task and limiting the efficacy of training. Consequently, approaches that do not account for graded sensory difficulty may not fully address the sensory demands experienced by individuals with CAI (Song et al., 2018). Therefore, the present findings provide a preliminary rationale for considering the gradual modification of sensory feedback as an adjunct to established rehabilitation, although its effects on postural control flexibility and functional stability require further investigation.

Secondly, the most severe instability occurred when both visual and somatosensory feedback were degraded simultaneously, with the CAI group experiencing up to a 180% decline. To address this issue and better mimic real-world challenges—such as navigating uneven ground in low light—rehabilitation protocols may be beneficial for integrate multi-sensory integration drills. Specifically, combining visual perturbation with unstable-surface training and immersive virtual reality may provide a structured way to progressively challenge multiple sensory pathways during rehabilitation (Kim et al., 2021; Lee et al., 2022b; Mohess et al., 2024). By layering sensory demands in a progressive manner, such approaches may warrant further investigation for their potential to promote more flexible postural control strategies, minimize over-reliance on any single sensory modality, and ultimately support more adaptable balance strategies in daily and athletic contexts involving complex sensory challenges.

### 4.5 Limitations and recommendations for future research

This study has some limitations. First, our sample comprised physically active young adults, which may limit the generalizability of the findings to other populations. Additionally, although the planned sample size of 26 participants per group was achieved, no additional participants were recruited to account for missing or unusable observations. We also did not include a coper group—individuals with a history of LAS but without ongoing symptoms or perceived ankle instability. Including coper group in future studies would allow for direct comparisons with CAI and healthy controls. This may offer valuable insights into protective or adaptive sensory organization strategies following LAS. Nevertheless, this study extends previous work by examining postural control responses across graded levels of individual and combined sensory disruption. Second, the flickering effects of the stroboscopic goggles may have induced additional visual disruption beyond visual occlusion alone. Future studies should consider luminance-controlled or VR-based visual manipulation to better isolate the effects of graded visual disruption. Third, each single-leg stance trial lasted 10 s. Although this duration is consistent with prior CAI single-leg balance studies and was selected to maintain task feasibility and data completeness under challenging sensory-disruption conditions, it remains unclear whether longer 20 to 30 s trials would produce similar findings. Fourth, although both groups received the same familiarization procedures and standardized rest periods, the performance-dependent number of repeated attempts resulted in greater trial exposure in the CAI group. Consequently, differential fatigue or learning effects between groups cannot be excluded. Fifth, the limb-selection procedure differed between groups: the involved or more symptomatic limb was tested in participants with CAI, whereas the preferred stabilizing limb was tested in healthy controls. Because only one limb was assessed, we could not isolate the potential influence of limb dominance or determine whether the observed group differences were affected by the limb-selection procedure. Lastly, this study assessed sensory organization indirectly through measures of postural control responses under sensory feedback disruption. Therefore, interpretations related to sensory reweighting should be considered inferential rather than direct evidence of altered central processing or neurophysiological mechanisms. Future studies using neurophysiological tools, such as electroencephalography (EEG) or transcranial magnetic stimulation (TMS) may help clarify how the CNS integrates multisensory inputs during postural control. EEG can reveal cortical activation patterns associated with sensory reweighting, while TMS may assess cortical excitability and inhibition in sensorimotor regions. A better understanding of these central mechanisms may help inform the development of neurosensory rehabilitation approaches for individuals with CAI.

## 5 Conclusion

The current study found that individuals with CAI and healthy controls showed broadly similar patterns of postural response to graded somatosensory and combined sensory disruption, although individuals with CAI demonstrated greater overall deterioration under several disrupted sensory conditions. Although individuals with CAI were particularly vulnerable under low-level visual disruption, both groups demonstrated similar adaptive responses to graded somatosensory challenge, while combined sensory disruption highlighted the exacerbated postural instability of CAI in multi-sensory environments. These findings suggest that individuals with CAI may retain some capacity to modify postural responses as sensory demands increase, although the underlying central mechanisms require further investigation. However, the interaction observed under visual disruption warrants further validation using controlled visual environments or virtual reality setups to clarify the level-dependent response pattern. Future research should also determine whether graded multisensory rehabilitation can improve functional stability in individuals with CAI across complex real-world settings.

## Data Availability

The raw data supporting the conclusions of this article will be made available by the authors, without undue reservation.
