# Supplementary material for: Postural control responses during single-leg balance in individuals with chronic ankle instability under graded visual and somatosensory disruption
Source: Front Psychol. 2026 Aug 13;17:1892350. doi: 10.3389/fpsyg.2026.1892350 (PMC13518158; doi:10.3389/fpsyg.2026.1892350)
Supplement: Supplementary file 1 [file Table_1.DOCX]

| COP parameters | 7 different levels of sensory disruption conditions | | | | | | | | |
| --- | --- | --- | --- | --- | --- | --- | --- | --- | --- |
|  | Reference Task | | Task 1  (visual disruption) | | Task2  (somatosensory disruption) | | Task3  (Combined visual-somatosensory disruption) | |  |
|  | Group | Condition 1 | Condition 2 | Condition 3 | Condition 4 | Condition 5 | Condition 6 | Condition 7 |  |
|  |  | Normal | VIS  Low | VIS  High | SOM  Low | SOM  High | VIS-SOM Low | VIS-SOM  High |  |
| Total Area (cm^2^) | CAI  CON | 3.84±2.70  2.82±2.39 | 8.87±6.63  5.74±4.83 | 7.51±5.01  6.24±5.48 | 5.58±3.88  3.64±3.13 | 6.43±4.61  4.25±3.58 | 12.83±9.24  8.86±7.45 | 16.38±11.77  11.38±9.55 |  |
| Total velocity (cm/s) | CAI  CON | 4.07±1.90  3.52±1.86 | 6.44±2.91  5.09±2.78 | 5.85±2.36  5.07±2.65 | 4.60±2.13  3.66±1.94 | 5.25±2.40  4.06±2.14 | 7.59±3.33  5.93±3.34 | 8.56±3.71  6.75±3.63 |  |
| Velocity-ML (cm/s) | CAI  CON | 2.69±1.39  2.40±1.28 | 4.00±1.89  3.30±1.77 | 3.68±1.64  3.34±1.74 | 3.00±1.42  2.44±1.24 | 3.44±1.62  2.73±1.46 | 4.68±2.19  3.60±1.91 | 5.32±2.41  4.28±2.25 |  |
| Velocity-AP (cm/s) | CAI  CON | 2.51±1.11  2.09±1.14 | 4.18±2.00  3.18±1.81 | 3.77±1.50  3.12±1.66 | 2.89±1.35  2.23±1.29 | 3.26±1.50  2.44±1.32 | 4.96±2.13  3.92±2.40 | 5.55±2.39  4.30±2.42 |  |

**Supplementary Table S1.** Center of pressure (COP) measures taken during single-leg stance for 10 seconds under different levels of sensory conditions.

Abbreviations: CAI, chronic ankle instability; CON, healthy controls; VIS, visual disruption; SOM, somatosensory disruption; VIS-SOM, combined visual-somatosensory disruption; ML, mediolateral; AP, anteroposterior.

Note. Values presented as mean ± standard deviation.

**Supplementary Table S2.** Comparison of original and covariate-adjusted analyses for group-by-sensory interaction and group main effects.

| Sensory  condition | COP parameters | Original  group-by- sensory Interaction | Covariate-adjusted group-by-sensory Interaction | Original group Main Effect | Covariate-adjusted  group main effect | Interpretation |
| --- | --- | --- | --- | --- | --- | --- |
| VIS | Total Area (%) | *F*=1.779, *p*=0.188 | *F*=1.563, *p*=0.218 | *F*=0.075, *p*=0.786 | *F*=1.102, *p*=0.299 | Maintained |
|  | Total velocity (%) | *F*=4.886, *p*=0.032**^†^** | *F*=5.058, *p*=0.029**^†^** | *F*=2.504, *p*=0.120 | *F*=3.144, *p*=0.083 | Maintained |
|  | Velocity-ML (%) | *F*=6.420, *p*=0.014**^†^** | *F*=6.427, *p*=0.015**^†^** | *F*=1.609, *p*=0.211 | *F*=2.907, *p*=0.095 | Maintained |
|  | Velocity-AP (%) | *F*=3.053, *p*=0.087 | *F*=3.153, *p*=0.082 | *F*=1.875, *p*=0.177 | *F*=2.057, *p*=0.158 | Maintained |
| SOM | Total Area (%) | *F*=0.096, *p*=0.758 | *F*=0.782, *p*=0.381 | *F*=4.357, *p*=0.042* | *F*=5.949, *p*=0.019* | Maintained |
|  | Total velocity (%) | *F*=2.176, *p*=0.146 | *F*=2.387, *p*=0.129 | *F*=6.009, *p*=0.018* | *F*=5.408, *p*=0.025* | Maintained |
|  | Velocity-ML (%) | *F*=2.181, *p*=0.146 | *F*=1.581, *p*=0.215 | *F* 6.259, *p*=0.016* | *F*=4.513, *p*=0.039* | Maintained |
|  | Velocity-AP (%) | *F*=1.540, *p*=0.220 | *F*=2.715, *p*=0.106 | *F*=2.904, *p*=0.095 | *F*=3.512, *p*=0.067 | Maintained |
| VIS+SOM | Total Area (%) | *F*=0.008, *p*=0.930 | *F*=0.060, *p*=0.808 | *F*=0.596, *p*=0.444 | *F*=1.847, *p*=0.181 | Maintained |
|  | Total velocity (%) | *F*=0.080, *p*=0.779 | *F*=0.069, *p*=0.794 | *F*=7.439, *p*=0.009* | *F*=9.592, *p*=0.003* | Maintained |
|  | Velocity-ML (%) | *F*=0.171, *p*=0.681 | *F*=0.025, *p*=0.874 | *F*=9.098, *p*=0.004* | *F*=10.211, *p*=0.003* | Maintained |
|  | Velocity-AP (%) | *F*=0.076, *p*=0.784 | *F*=0.151, *p*=0.700 | *F*=3.294, *p*=0.076 | *F*=4.811, *p*=0.033* | Significant group main effect |

Abbreviations: AP, anteroposterior; COP, center of pressure; ML, mediolateral; SOM, somatosensory condition; VIS, visual condition.

Note. Covariate-adjusted analyses were performed using repeated-measures general linear models adjusted for age, height, body mass, and physical activity level, as assessed using the Godin Leisure-Time Exercise questionnaire.

^†^ Indicates a significant interaction between group and sensory disruption level (*p*<0.05).

* Indicates a significant group main effect (*p*<0.05).
